# Development and applications of a collection of single copy gene-based cytogenetic DNA markers in garden asparagus

**DOI:** 10.3389/fpls.2022.1010664

**Published:** 2022-09-29

**Authors:** Chen You, Ruidong Wen, Zhilong Zhang, Guangqian Cheng, Yulan Zhang, Ning Li, Chuanliang Deng, Shufen Li, Wujun Gao

**Affiliations:** College of Life Sciences, Henan Normal University, Xinxiang, China

**Keywords:** chromosome identification, *Asparagus officinalis*, single copy gene, fluorescence *in situ* hybridization (FISH), molecular cytogenetic analysis

## Abstract

Garden asparagus (*Asparagus officinalis*, 2n = 2x = 20 chromosomes) is an important dioecious vegetable crop and a model species for studying sex chromosome formation and evolution. However, few molecular cytogenetic studies on garden asparagus have been reported because of its small metaphase chromosomes, the scarcity of distinguished cytogenetic markers, and the high content of repetitive sequences. In this study, a set of single copy genes free of repetitive sequences with sizes ranging from 4.3 kb to 8.2 kb were screened and used as probes for fluorescence *in situ* hybridization (FISH) to identify individual chromosomes of garden asparagus. The chromosome-specific signal distribution patterns of these probes enabled the distinguishment of each pair of chromosomes. The sequence assembly and cytogenetic map were successfully integrated, and the results confirmed that the chromosome 1 representing the sex chromosome in the genome assembly is chromosome 5 in the karyotype analysis. The cytogenetic identification of the male-specific region of the Y chromosome (MSY) was implemented using a mixed probe derived from a number of MSY-specific single copy sequences. In addition, the chromosome orthologous relationship between garden asparagus (A1–A10, karyotypic analysis) and its hermaphrodite close relative, *A. setaceus* (B1–B10, karyotypic analysis), was analyzed using this collection of chromosome-specific cytological markers. The results showed that B3 is the ortholog of sex chromosome A5 and thus may represent the ancestral autosome of the current sex chromosome in garden asparagus. Chromosomes B5, B4, B1, B8, B7, and B9 are the orthologs of A2, A3, A4, A7, A8, and A10, respectively. The chromosome identification, cytogenetic recognition of MSY, and the orthologous relationship analysis between garden asparagus and *A. setaceus* are valuable for the further investigation of the sex chromosome emergence and evolutionary mechanism of garden asparagus and genome structure evolution in the *Asparagus* genus.

## Introduction

Fluorescence *in situ* hybridization (FISH), which enables the intuitive detection of specific DNA sequences on chromosomes, plays a significant role in plant molecular cytogenetic research (Jiang and Gill, 1994; Jiang and Gill, 2006; Zhang et al., 2016). The FISH-based molecular cytogenetic study provides powerful tools for visualization, genetics, epigenetic, and cytological clues that can be used as important supplement for molecular genomic research. It can be utilized for chromosome identification (Hizume et al., 2002), karyotype construction (Li et al., 2019), physical mapping of DNA sequences (Kirov et al., 2017), chromosome pairing tracing (Lee et al., 2011), and chromosome rearrangement and evolution study (Divashuk et al., 2014; Lou et al., 2014; Mandakova et al., 2019). For example, molecular cytogenetic analysis combined with conventional cytogenetic and immunofluorescence assays unraveled the knob-like heterochromatin structures characteristic to the male-specific region of the Y chromosome (MSY) of papaya and suggested the important role of DNA methylation and heterochromatinization in the early stage of sex chromosome evolution (Zhang et al., 2008).

Garden asparagus (*Asparagus officinalis*, 2n = 2x =20 chromosomes) is an important vegetable crop that has great nutritional and economic values (Fan et al., 2015; Witzel and Matros, 2020). It is also a dioecious species with genders governed by a pair of homomorphic X and Y chromosomes. In addition, it has hermaphrodite close relatives within the same genus, *Asparagus*, such as *A. setaceus*. Thus, garden asparagus offers an ideal system for studying sex chromosome emergence and evolution (Arumuganathan and Earle, 1991; Li et al., 2017). Extensive studies on this species can lay a foundation for the improvement of its breeding and cultivation and can also shed light on the early stages of sex chromosome evolution. Conventional cytogenetic studies using chromosome banding technique showed that the 10 chromosomes of garden asparagus can be classified as long (L1–L5), medium (M1), and short (S1–S4) chromosomes according to their size (Löptien, 1976). Primary trisomics analysis revealed that chromosome L5 is the sex chromosome harboring the sex locus (Löptien, 1979). DNA sequence-based high-throughput technologies have been recently carried out on this species widely and achieved great progress (Harkess et al., 2017; Li et al., 2021). For example, the assembly of the garden asparagus “supermale” genome, which is 986 Mb, can be assigned to 10 chromosomes. An 847 kb MSY was identified on the Y chromosome. Within the MSY, one male-promoting and one female-suppressing genes were detected, and functional analysis confirmed that these two genes are sex-determining genes (Harkess et al., 2020). Moreover, DNA methylation plays important roles in sexual differentiation and sex chromosome evolution in this species (Li et al., 2021). Besides, many sex-biased genes that potentially played roles in sexual differentiation and reproductive organ development process have been identified (Harkess et al., 2015; Li et al., 2017).

For molecular cytogenetic studies on garden asparagus, several research groups applied ribosomal DNA genes (45S and 5S rDNA) as FISH probes for chromosome identification and karyotyping. However, except for rDNA localization, few molecular cytogenetic studies on garden asparagus have been reported. This is largely because of its small metaphase chromosomes and a high content of repetitive sequences (over 70% of the genome) (Telgmann-Rauber et al., 2007; Harkess et al., 2017). Although 5S and 45S rDNAs were mapped on one and three pairs of chromosomes, respectively (Reamon-Buttner et al., 1999; Melo and Guerra, 2001; Deng et al., 2012; Mousavizadeh et al., 2016), the information on rDNA locations is insufficient or inaccurate for the identification of all chromosomes in garden asparagus. In addition, different karyotypical studies on garden asparagus showed controversial results. For example, it has been reported that the L5 chromosome has 45S rDNA loci (Deng et al., 2012), whereas another study revealed that the sex-determining L5 chromosome harbors 5S rDNA loci (Moreno et al., 2018). Thus, the chromosome identification and karyotype of garden asparagus are still elusive. Molecular cytogenetic studies on this dioecious plant can provide new insights into its chromosome structure and evolution.

In this study, FISH analysis based on single copy genes was used to promote molecular cytogenetic studies in these dioecious plants. The aims of this study were to: i) develop a FISH system using single copy genes in garden asparagus; ii) construct a set of chromosome-specific cytogenetic markers to identify individual chromosomes and facilitate karyotyping; iii) integrate genome assembly and cytogenetic mapping; iv) identify MSY cytogenetically using MSY-specific probes; v) analyze the chromosome orthologous relationship between garden asparagus and its hermaphrodite close relative, *A. setaceus*, using the set of chromosome-specific cytological markers.

## Materials and methods

### Plant materials

Garden asparagus cv. ‘UC309’ grown in an experimental field at Henan Normal University and *A. setaceus* cv. ‘Pyramidalis’ cultivated in the glasshouse were used in this study. The seeds were harvested in autumn and stored at 4°C until use. Total DNA was isolated from the young cladodes of each species using the classical CTAB method.

### Identification of single copy genes free of repetitive sequence

The garden asparagus genome (Aspof.V1) and related annotation files were downloaded from the National Center for Biotechnology Information for the designing of single copy gene probes. First, the repetitive sequences of the garden asparagus genome were annotated using RepeatModeler (version 1.0.10) and LTRharvest (Ellinghaus et al., 2008) to eliminate the interference of repetitive sequences. The garden asparagus genome was initially masked using RepeatMasker (version 4.0.7) with the library generated by RepeatModeler, and the unmasked sequences were further masked by the library generated by LTRharvest. Next, the genome sequences of all the annotated genes were extracted from the annotation file. The genes with a sequence length shorter than 4000 bp or repetitive sequences masked more than 10% of the sequences were removed. Finally, a BLAST analysis of the garden asparagus gene database was conducted to eliminate genes with multiple copies. The single copy gene sequences were used for primer design using Premier 6.0 and Oligo 7 software, and the flanking sequences free of repetitive sequences were included if needed. The gene sequences or genes with flanking sequences were dissected into several segments for primer design to facilitate the amplification. For the probes designed to localize the MSY, the 847 kb sequences of MSY were extracted and masked. A number of single copy sequences free of repetitive sequences were identified and used for primer design. The primers used for amplifying single copy genes and single copy sequences in MSY are presented in **Table S1**. The designed primers were used for PCR amplification. After electrophoresis, products with expected size were cut from the gel and purified using a gel recovery kit (TransGen, Beijing, China), and then the concentration and purity of the purified product were measured by spectrophotometry.

### Probe labeling

The amplified and purified DNAs were labeled with Texas-red-dCTP (PerkinElmer, Waltham, Massachusetts, USA) by adopting the nick translation procedure used previously (Birchler et al., 2007). The 45S rDNA was also labeled with Chroma Tide Alexa Fluor 488-5-dUTP [Thermo Fisher Scientific (Invitrogen), Waltham, MA, USA] for FISH analysis.

### Preparation of genome-blocking DNAs

Genome-blocking DNAs for FISH were prepared to reduce the interference of repetitive sequences on single copy probe signals. Briefly, garden asparagus genomic DNA was diluted to 2000 ng/µL using double-distilled H_2_O. Then the DNA was sheared by incubating the tube containing the DNA sample in boiling water. The sheared DNA was then cooled in ice water for 1 min, followed by incubation in a water bath at 65°C for 4 min and 10 sec for reannealing. Renaturation time was calculated according to the formula: Cot = 1 = *C* (mol/L) × *T*_s_ (Britten and Kohne, 1968). The ideal genome-blocking DNAs were about 500 bp in length, and the concentration was adjusted about to 1500 ng/μL.

### Preparation of chromosome slides

The garden asparagus and *A. setaceus* seeds were cultivated in an incubator maintained at about 25°C under dark conditions. After 4–5 days, when the roots grew to about 1 cm in length, they were excised and treated with 1.01 MPa N_2_O for 1.5–2 h, fixed with 90% glacial acetic acid for about 10 min, and stored in 70% ethanol at –20°C. Before enzyme digestion, the root tips were taken out from the refrigerator and washed three times in 1 × citric buffer. The meristematic zone of the root tip was cut off and immersed in 20 µL of 1% pectolyase Y-23 (Yakult Pharmaceutical, Tokyo, Japan) and 2% cellulose Onozula R10 (Yakult Pharmaceutical) for 2 h at 37°C. Then, the samples were washed with 70% anhydrous ethanol and uniformly crushed. The solution was centrifuged at 1500 g for 60 s, and the supernatant was discarded. Then, the precipitate was mixed well with 35 µL of glacial acetic acid. Finally, the suspension was dropped onto a glass slide for later use.

For meiotic chromosome preparations, young flower buds with a length of 0.8–1 mm were placed in a fixed solution of acetic acid/ethanol (3:1) for 24 h. Anthers were extracted from immature flower buds for enzymatic hydrolysis. The meiotic preparations were prepared using the same method used to prepare the metaphase preparations described above.

### FISH analysis

FISH experiment was conducted as described previously (Li et al., 2019) with minor modifications. Chromosome slides were placed in an ultraviolet crosslinker for 2 min. The hybridization mixture was prepared by adding 100 ng/µL 45S rDNA probe 0.5 µL, single copy gene probes 3.5 µL (total amount of 1000 ng), 1500 ng/µL blocking DNA and 2 × SSC + 1 × TE buffer into the centrifugal tube and mixed well. In order to gain clear signal with low background, we optimized the ratio of blocking DNA to probe (**Table S3**). The volumes of blocking DNA and 2 × SSC + 1 × TE buffer for hybridization mixture varied according to the ratio of blocking DNA to probe. Then the hybridization mixture was added to the slide, and covered with a coverslip. The slides were put in a metal tray, boiled for 5 min, and then placed in a humid chamber at 55 °C for 12 h. After hybridization, the slides were washed three times in 2 × SSC and then counterstained with 4′,6-diamidino-2-phenylindole (Vectashield mounting media with DAPI, Vector Laboratories, Burlingame, USA). The images were captured with an Olympus BX63 fluorescence microscope using an ANDOR charge-coupled device with a 130 W long-lived fluorescence light source. The filters were as following: Chroma Tide Alexa Fluor 488 (exciting light 472 ± 30 nm, emission light 520 ± 35 nm), Texas-red (exciting light 543 ± 22 nm, emission light 593 ± 40 nm) and DAPI (exciting light 387 ± 11 nm, emission light 447 ± 60 nm). Finally, Photoshop CS6 was used to process the image.

## Results

### Identification of repeat-free single copy genes in the genome of garden asparagus

**Figure 1:**
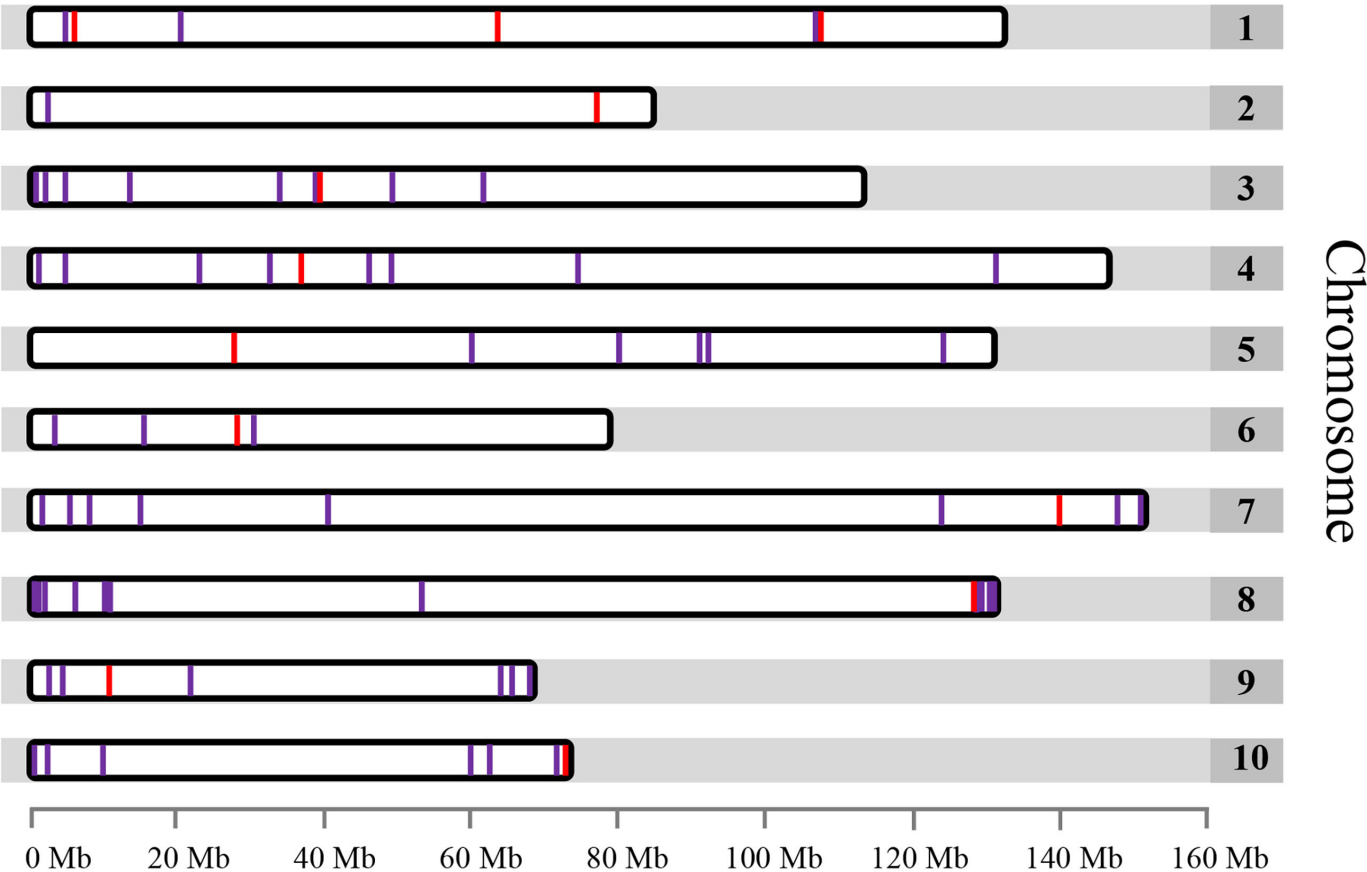
Distribution of the 71 identified single copy genes free of repetitive sequence within the garden asparagus genome represented by 10 chromosomes. Only single copy genes located on chromosomes are shown. The red lines denote the single copy genes that were used for FISH.

To develop chromosome-specific cytogenetic markers using FISH, the whole genome of garden asparagus was analyzed to identify single copy genes without repetitive sequences. Seventy-one single copy genes free of repetitive sequences were identified after the screening (**Table S2**). These genes were unevenly distributed on each chromosome, ranging from only 3 genes on chromosome 10 to 12 genes on chromosome 2 of the genome assembly (**Figure 1**, **Table S2**). Remarkably, most of the single copy genes were located at the two terminals of each chromosome (**Figure 1**).

### Establishment of single copy gene-based FISH technique for garden asparagus

**Figure 2:**
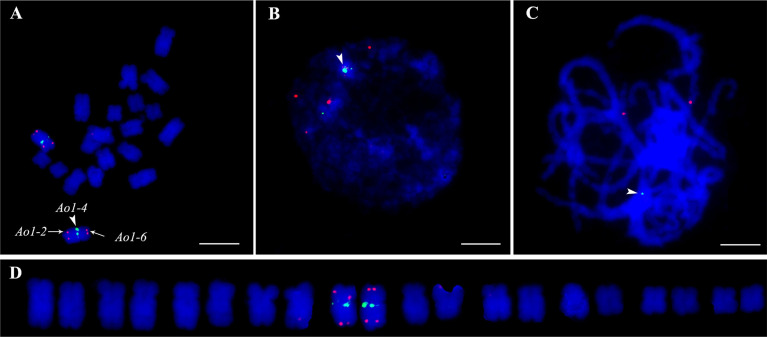
Accuracy validation of single copy gene-based FISH protocol in garden asparagus using three gene probe cocktail. The probes corresponding to *Ao1-2*, *Ao1-4*, and *Ao1-6* genes, which are all located on chromosome 5, were co-hybridized onto chromosome 5 at metaphase **(A)**, interphase **(B)**, and meiotic pachytene **(C, D)** Karyotypic analysis. The red signals of the *Ao1-2* and *Ao1-6* probes are at the end of the long and short arms of chromosome 5, respectively, whereas the green signals of the *Ao1-4* probe are at the centromeres of chromosome 5. Scale bar = 10 μm.

A reliable single copy gene-based FISH protocol was developed for garden asparagus, whose genome harbors plenty of repetitive sequences. The ratio of blocking DNA to probe in the hybridization procedure was optimized to acquire clearer FISH signals in garden asparagus (**Table S3**). The result showed that the background noises were gradually attenuated along with the increased ratio of blocking DNA to probe. The background signal was hardly detected when the ratio value was equal to or greater than 6:1 (**Figure S1**). Three genes (designated as *Ao1-2*, *Ao1-4*, and *Ao1-6*) located on chromosome 1 of the genome assembly were labeled with Texas red or Chroma Tide Alexa Fluor 488 to test the reliability of the single copy gene FISH protocol. *Ao1-2*, *Ao1-4*, and *Ao1-6* had probe lengths of 5848, 5772, and 4378 bp, respectively, and located at 6.5, 64.2, and 109 Mb, respectively. The probes were co-hybridized with chromosomes at mitotic metaphase, interphase, and meiotic pachytene stage (**Figure 2**). Consistent with the location of the three genes in the genome assembly, the red hybridization signals of *Ao1-2* and *Ao1-6* were concurrently present at the ends of the long and short arms of chromosome 5, respectively, and the green hybridization signals for *Ao1-4* were present at the centromeres of chromosome 5 (**Figure 2**). Collectively, the single copy gene FISH protocol with sufficient sensitivity and reliability can fulfill chromosome identification and other applications.

### Identification of individual chromosomes by single copy cytogenetic markers in garden asparagus

**Figure 3:**
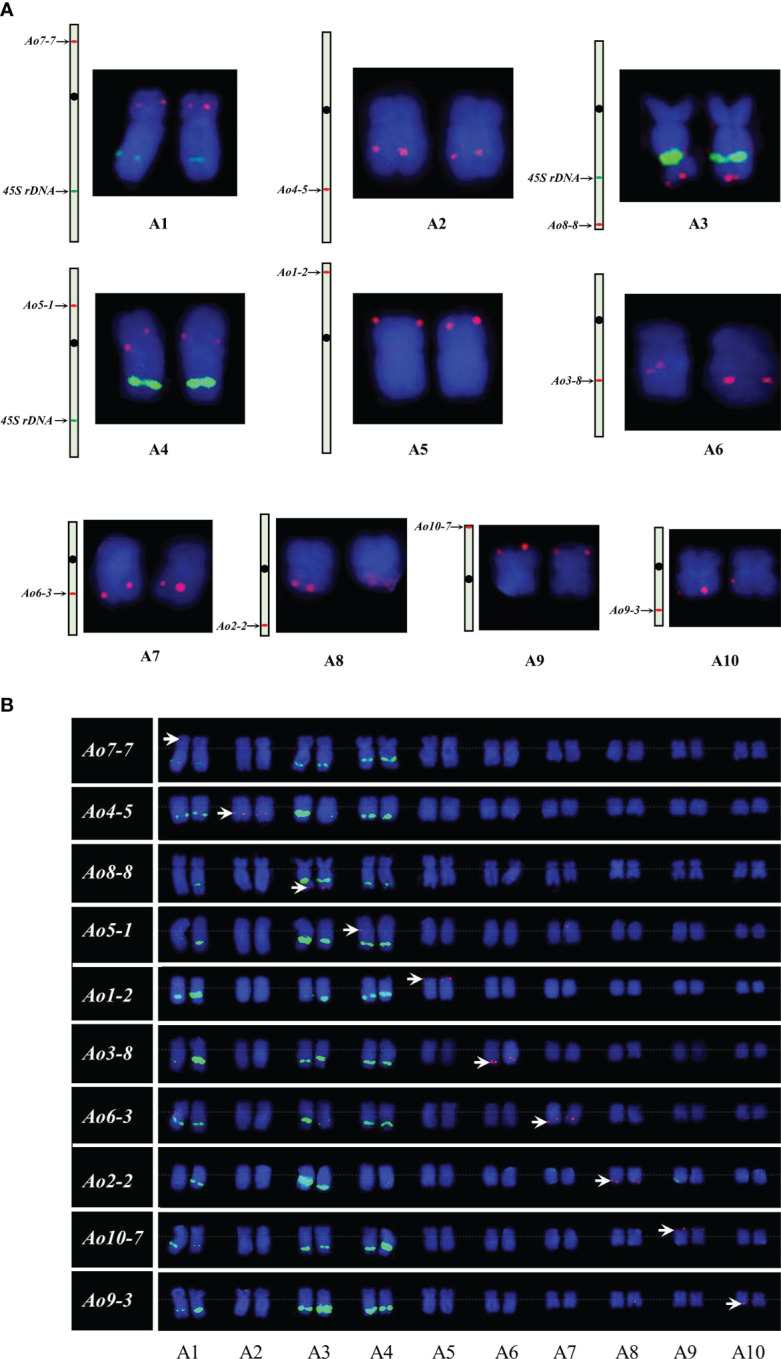
Single copy gene-based FISH analysis on somatic chromosomes in garden asparagus. **(A)** Sequence mapping of chromosome-specific FISH probes and their FISH signals on individual chromosomes. **(B)** Karyotypic analysis of garden asparagus chromosome based on chromosome-specific markers. The arrows denote the chromosome-specific FISH signal (red). The green signal represents the signals of 45S rDNA.

**Figure 4:**
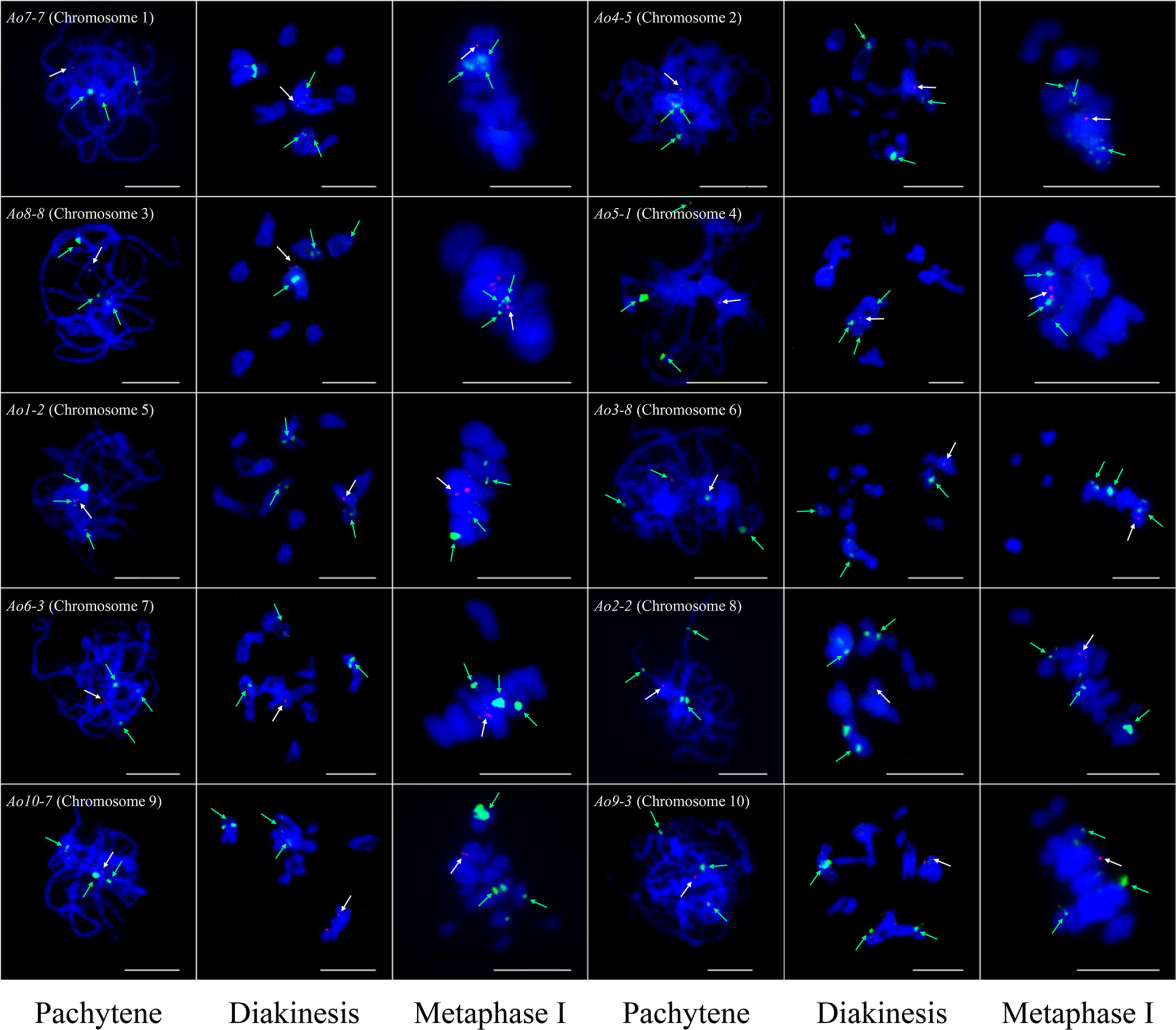
Single copy gene-based FISH analysis on meiotic chromosomes at different stages in garden asparagus. The white arrows denote the chromosome-specific FISH signal (red). The green arrows denote the signals of 45S rDNA (green). Bars = 10 μm.

At least one single copy gene was selected as the FISH probe for the identification of each individual chromosome pair. These single copy genes had different sizes within the range of 4.3–8.2 kb. For example, *Ao7-7* located on chromosome 7 of the genome assembly was used to identify chromosome 1 in the karyotypic analysis at different cell cycle phases (**Figures 3** and **4**). Thus, a collection of chromosome-specific cytogenetic markers was developed and used with 45S rDNA to identify all 10 chromosome pairs and construct the garden asparagus karyotype (**Table 1**; **Figure 3**). The signal positions of each gene probe were in accordance with their corresponding gene locations on the sequence map (**Figure 3**). In addition, the probes were all hybridized on meiotic chromosomes, and the results confirmed that these probes could provide reliable chromosome-specific cytogenetic markers (**Figure 4**). The genome assembly and cytogenetic map could be integrated using this collection of chromosome-specific markers, and each chromosome of the genome assembly matched one chromosome in the karyotypic analysis. That is, the chromosomes 1–10 of the genome assembly corresponded to chromosomes 5, 8, 6, 3, 4, 7, 1, 2, 10, and 9 in the karyotypic analysis, respectively. Specifically, the chromosome 1 representing the sex chromosome in the genome assembly corresponded to chromosome 5 in the karyotype analysis (**Figure 3**; **Table 1**).

**Table 1:** The information of single copy genes used for chromosome identification in garden asparagus.

Serial number	Gene name	Length (bp)	Chromosome	Start	End
LOC109848092	*Ao7-7*	7316	1	139581277	139573962	
LOC109837191	*Ao4-5*	4683	2	36621730	36626412	
LOC109851190	*Ao8-8*	7168	3	128118248	128111081	
LOC109841951	*Ao5-1*	8021	4	27970139	27978159	
LOC109847450	*Ao1-2*	5848	5	6509204	6503357	
LOC109825211	*Ao1-4*	5772	5	64286784	64281013	
LOC109828924	*Ao1-6*	4378	5	109045790	109041413	
LOC109833867	*Ao3-8*	5207	6	38951082	38956288	
LOC109845972	*Ao6-3*	6386	7	28335011	28341396	
LOC109831691	*Ao2-2*	8645	8	77443879	77452523	
LOC109825143	*Ao10-7*	8226	9	72435441	72443666	
LOC109824012	*Ao9-3*	8109	10	10773569	10781677	

### Cytogenetic identification of MSY in garden asparagus

**Figure 5:**
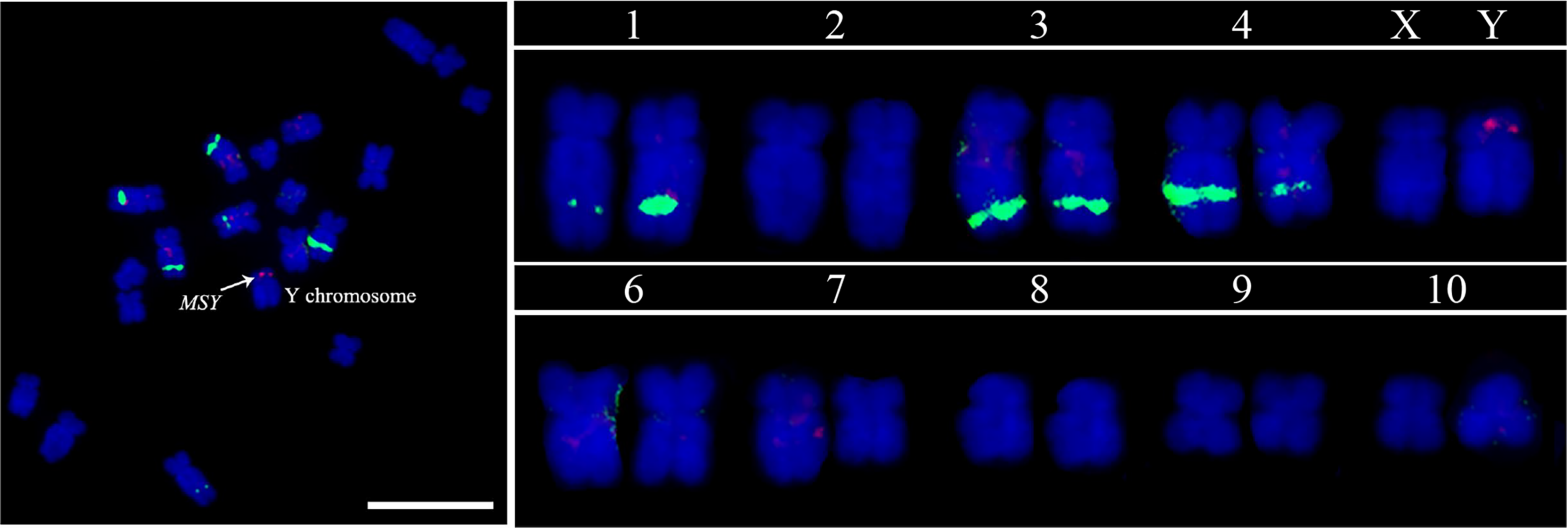
MSY identification based on FISH analysis using five MSY-specific single copy sequence probe cocktails. The red signal denotes the MSY, and the green signals indicate the 45S rDNA. Scale bar = 10 μm.

RepeatMasker analysis showed that the MSY was enriched abundant transposable elements (86.95%). The 847 kb MSY sequences were masked and subjected to BLAST against the whole garden asparagus genome, and five single copy sequences in MSY were identified, namely, *msy-2* (877 bp), *msy-6* (621 bp), *msy-9* (1400 bp), *msy-10* (1446 bp), and *msy-12* (2468 bp). A mixture of the five single copy sequences was used to visualize MSY at mitotic metaphase (**Figure 5**). The FISH result showed that a bright red fluorescent signal was at the end of the short arm of the Y chromosome, whereas no signal was found on the X chromosome (**Figure 5**).

### Mis-assembly of the genomic sequence is detected by the single copy gene-based FISH

**Figure 6:**
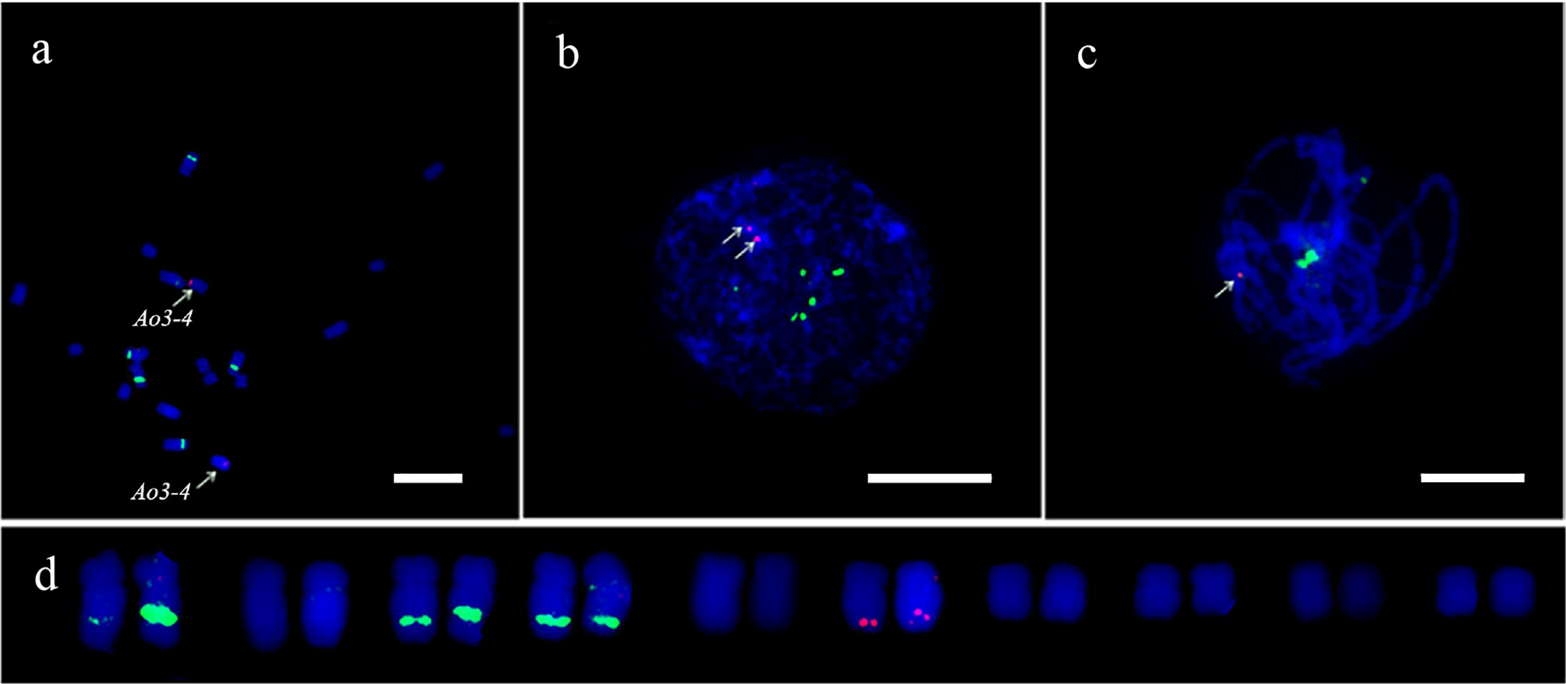
FISH analysis of the *Ao3-4* gene to evaluate the quality of the sequence assembly. FISH mapping of the *Ao3-4* gene using the 5 kb probe (red) and 45S rDNA probe (green) at mitotic metaphase **(A)**, interphase **(B)**, and meiotic pachytene **(C, D)** The karyotypic analysis. Scale bar = 10 μm.

In this study, FISH was used to check the authenticity of a three-copy gene, *Ao3-4*. In the genome assembly, one copy of *Ao3-4* was located on chromosome 3 (NC_033796.1) and the other two copies were located on unplaced scaffolds. Instead of three pairs of expected fluorescent signals, only one pair of bright signals were detected at the terminus of the long arm at mitotic metaphase and interphase, as well as at meiosis pachytene (**Figure 6**). This unexpected FISH result may be due to mis-assembly of the *Ao3-4* gene.

### Orthologous relationship analysis between garden asparagus and *Asparagus setaceus*

**Figure 7:**
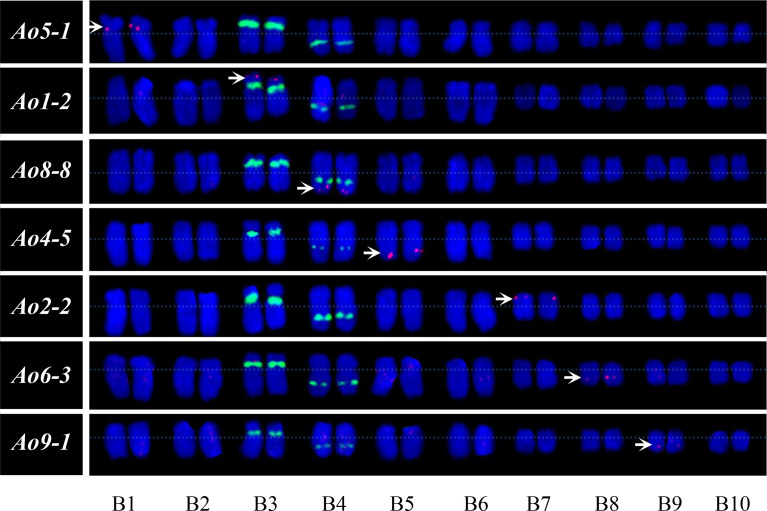
Orthologous relationship analysis and karyotype of *A. setaceus* based on FISH analysis using garden asparagus chromosome-specific single copy genes. The arrows denote the chromosome-specific FISH signal (red). The green fluorescence represents the signals of 45S rDNA.

The garden asparagus chromosome-specific single copy gene probes hybridized to the metaphase chromosomes of *A. setaceus* were used to examine whether the garden asparagus probes generate detectable signals of the chromosomes of its hermaphrodite close relative, *A. setaceus*, and to reveal their homoeologous relationships. *Ao3-8, Ao7-7*, and *Ao10-7* did not generate any signal in our experiment, and the remaining seven of the 10 probes could generate clear and reproducible signals. The karyotypic chromosomes of garden asparagus and *A. setaceus* were designated as A1–A10 and B1–B10, respectively. The FISH analysis revealed that B3 is the ortholog of sex chromosome A5 and thus represents the ancestral autosome of the current sex chromosome in garden asparagus. Moreover, chromosomes B5, B4, B1, B8, B7, and B9 are the orthologs of A2, A3, A4, A7, A8, and A10, respectively (**Figure 7**).

## Discussion

FISH is a cytogenetic tool extensively used to examine many aspects associated with chromosomes. The development of probes that show chromosome-specific labeling patterns is of vital importance to ensure a successful FISH analysis. Repetitive DNA sequences, especially satellites, are usually served as FISH probes owing to their specific FISH signal patterns on distinctive chromosomes within a given species (Schneider et al., 2003; He et al., 2015; Tang et al., 2016; Li et al., 2019). The garden asparagus genome was also scanned for the detection of satellites that can be used as probes potential suitable for chromosome identification. However, no high-confidence tandem repeat was identified in the garden asparagus genome using unassembled reads with the TAREAN online pipeline (Novak et al., 2017; Our unpublished results). Moreover, FISH using single copy genes as probes can be used more widely than that using repetitive sequences as probes, such that it can also be used for cross-species orthology analysis (Danilova et al., 2012). However, FISH using single copy genes as probes is more difficult than that using repetitive sequences as probes (Wang et al., 2006). Thus, single copy gene-based FISH analysis is only used in several species, such as those in *Triticum* (Danilova et al., 2012; Danilova et al., 2014), maize (Wang et al., 2006; Lamb et al., 2007), and cucumber (Lou et al., 2014). In this study, single copy gene FISH was established in the dioecious plant, garden asparagus. Through the optimization of the probe preparation system and the ratio of blocking DNA to probe, the FISH technique based on single copy genes is sensitive enough to detect the probes of about 4 kb size in the metaphase chromosome of garden asparagus. This is less sensitive than that used in cucumber (Lou et al., 2014) and similar to those used in maize (Wang et al., 2006) and rice (Jiang et al., 1996). This result may be due to the high repeat content and chromatin features of the garden asparagus genome. Based on this technique, a set of chromosome-specific cytogenetic markers covering all the chromosomes in the garden asparagus complement were designed and used for chromosome identification, integration of sequence assembly and cytogenetic map, MSY localization, genome assembly evaluation, and the orthologous relationship analysis between garden asparagus and its relative. Furthermore, the single copy gene based FISH technique can also be introduced to other species, and have a number of applications, including all the applications used in the garden asparagus.

Each pair of chromosomes in garden asparagus can be easily identified using this set of cytogenetic markers developed by utilizing single copy genes free of repetitive sequences as probes for FISH analysis. It can be used for chromosome traceability in garden asparagus, as well as in the construction of garden asparagus karyotypes. Unfortunately, a maximum of three probes can be mapped in one FISH experiment owing to the limitations of probe mix volume and probe concentration. Thus, each pair of chromosomes was not identified in one experiment in this study. Nevertheless, sufficient information about the karyotype of garden asparagus could be obtained from the FISH analysis of single copy genes combined with 45S rDNA. The 45S rDNA showed signals on chromosomes 1, 3, and 4, whereas the sex chromosomes are in chromosome 5 with no 45S rDNA locus in the karyotype. This result was in contrast to the previous results that chromosome 5 has 45S rDNA loci (Deng et al., 2012). Sequence assembly and cytogenetic map were also integrated in the present study. Each chromosome in the sequence assembly matched a chromosome in the karyotypic analysis. We also used a three copy gene, *Ao3-4*, from the genome assembly as a probe, and FISH analysis showed that only one pair of signals exited both at interphase and metaphase stages. This result suggested that this gene may be a single copy gene. Alternatively, this gene may have three copies within a 100 kb region, for that the resolution of interphase FISH is 50−100 kb (Jiang et al., 1996). Considering that the signals are small and sharp, this gene is more likely a single copy gene. Thus, the three copies in the genome annotation may be due to a mis-assembly of the corresponding region. Furthermore, the MSY region was cytologically detected. This means that the Y chromosome harboring MSY was also cytologically identified. In the early literatures, chromosome L5 (the fifth long chromosome) was believed to be the sex chromosome harboring the sex locus (Löptien, 1979). However, for a quite long time, which chromosome is L5 confused us, and different groups showed different results (Deng et al., 2012; Moreno et al., 2018), mainly because of the similar size of the first five pairs of chromosomes. Here, we utilized the sequences of the MSY region as a probe to detect the MSY and Y chromosome, and this result is more reliable than that used previously (Deng et al., 2012; Moreno et al., 2018). These cytological advances facilitate further studies on cytogenetic characterization of chromosome structure, especially on cytogenetic study of sex chromosome structure and evolution.

FISH can also be used for cross-species analysis, which can offer intuitive evidence for sequence conversation among related species. Previously, cross-species FISH analysis has been conducted in a number of plant species (Lysak et al., 2006; Tang et al., 2008; Wolny et al., 2013; Wang et al., 2017). For example, cross-species FISH based on full-length cDNAs was applied to detect chromosomal rearrangement in wheat and wild Triticeae species (Danilova et al., 2014). In this research, seven single copy gene probes from garden asparagus were localized on *A. setaceus* chromosomes successfully, and the chromosomal orthologous relationships between the two relatives could be analyzed according to FISH signals. The results showed that B3 is the ortholog of sex chromosome A5 and thus represents the ancestral autosome of the current sex chromosome in garden asparagus. Further cytogenetic analysis of these two chromosomes, such as heterochromatin analysis, could provide supplementary information on the sex chromosome evolution of garden asparagus.

## Conclusions

A reliable single copy gene FISH technology was developed and used for garden asparagus molecular cytogenetic characterization. A collection of chromosome-specific cytogenetic markers was developed, and they can be used for chromosome identification, genome assembly and cytogenetic map integration, and chromosome orthologous relationship analysis between asparagus and its relatives. In addition, a mix of single copy sequence probe was also used to cytogenetically detect MSY. This study paves a way for further studies regarding the cytogenetic demonstration and sex chromosome evolutionary mechanism of garden asparagus. It also facilitates the study of chromosome structure evolution in the *Asparagus* genus.

## Data availability statement

The original contributions presented in the study are included in the article/**Supplementary Material**. Further inquiries can be directed to the corresponding authors.

## Author contributions

SL and WG designed the experiments. CY, RW, ZZ, and GC conducted the study and processed the data. CY and SL wrote the manuscript. CY, YZ, NL, CD, SL and WG discussed the results and revised the manuscript. All authors contributed to the article and approved the submitted version.

## Funding

This work was financially supported by the National Natural Science Foundation of China (31970240 and 32170336), the Natural Science Foundation of Henan province (222300420053), and the Program for Science and Technology Innovation Talents in the Universities of Henan Province (23HASTIT035).

## Acknowledgments

We thank all members who participated in this study.

## Conflict of interest

The authors declare that the research was conducted in the absence of any commercial or financial relationships that could be construed as a potential conflict of interest.

## Publisher’s note

All claims expressed in this article are solely those of the authors and do not necessarily represent those of their affiliated organizations, or those of the publisher, the editors and the reviewers. Any product that may be evaluated in this article, or claim that may be made by its manufacturer, is not guaranteed or endorsed by the publisher.

## Supplementary material

Supplementary Figure 1 Effect of blocking DNA on hybridization results using the *Ao8-8* probe. **(A–D)** Hybridization results with different ratios of blocking DNA to the *Ao8-8* probe. The red signal on the chromosome long arm is the *Ao8-8* hybridization signal, and the green signal is the 45S rDNA hybridization signal. Scale bar = 10 μm.
