# Supplementary figures and images for: Development and applications of a collection of single copy gene-based cytogenetic DNA markers in garden asparagus
Source: Front Plant Sci. 2022 Sep 29;13:1010664. doi: 10.3389/fpls.2022.1010664 (PMC9559582; doi:10.3389/fpls.2022.1010664)

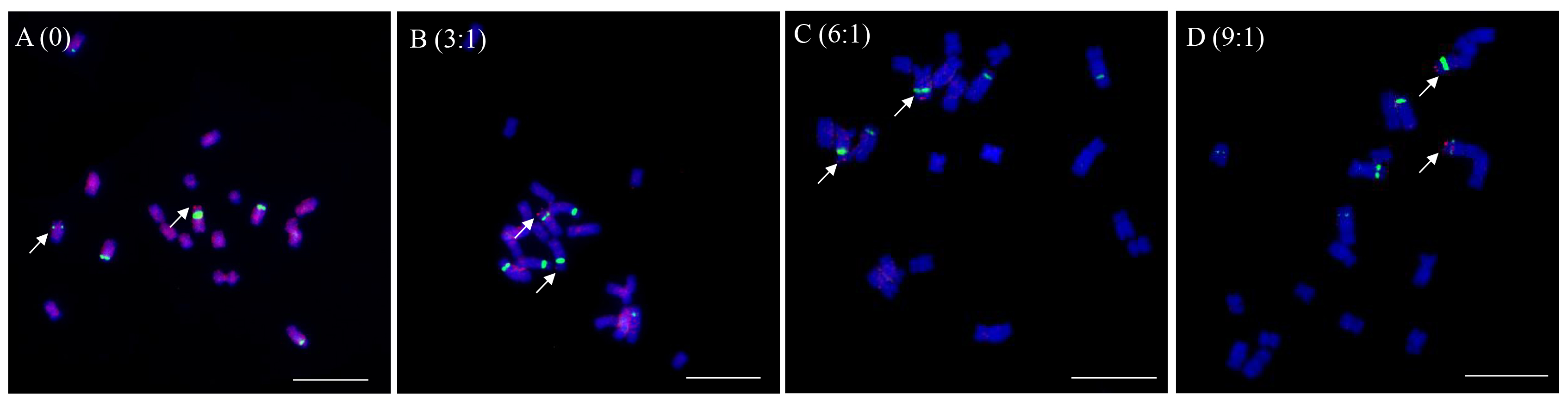

Supplement: Supplementary Figure 1 — Effect of blocking DNA on hybridization results using the Ao8-8 probe. (A–D) Hybridization results with different ratios of blocking DNA to the Ao8-8 probe. The red signal on the chromosome long arm is the Ao8-8 hybridization signal, and the green signal is the 45S rDNA hybridization signal. Scale bar = 10 μm. [file Image_1.jpeg]
