# Supplementary material for: Development and applications of a collection of single copy gene-based cytogenetic DNA markers in garden asparagus
Source: Front Plant Sci. 2022 Sep 29;13:1010664. doi: 10.3389/fpls.2022.1010664 (PMC9559582; doi:10.3389/fpls.2022.1010664)
Supplement: Supplementary file 2 [file Table_1.docx]

Table S1. The list of primers used in this study.

| Primer name | Sequence (5′-3′) | Annealing temperature (℃) |
| --- | --- | --- |
| *Ao1-2F* | GTTTCCCGTTGATCCGAACCTACCT | 59 |
| *Ao1-2R* | ATCCAACGGCAGCAACTTCAGTATG |  |
| *Ao1-2-1F* | CCATTAGATTCCAGTTCACCTT | 56 |
| *Ao1-2-1R* | AGGGATGAAGAATATGGAGCAG |  |
| *Ao1-2-2F* | CTGCTCCATATTCTTCATCCCT | 56 |
| *Ao1-2-2R* | CTAAATGCCAAACAATCTGCTT |  |
| *Ao1-2-3F* | CTCAACCTTTTATTATTGGGAC | 57 |
| *Ao1-2-3R* | TTCCATAAGTTGATGACTGCAA |  |
| *Ao1-2-4F* | TTGCAGTCATCAACTTATGGAA | 56 |
| *Ao1-2-4R* | AGGTGAACTGGAATCTAATGGG |  |
| *Ao1-4-1F* | TTGGTAACCTGTGAACTAAGCC | 57 |
| *Ao1-4-1R* | ATTCACCTGATTGCTTGCGCTA |  |
| *Ao1-4-2F* | GTCAACACACAGGCGTCGGGAA | 57 |
| *Ao1-4-2R* | TAATGGGTAGTGGGAGATTCGG |  |
| *Ao1-4-3F* | CCGAATCTCCCACTACCCATTA | 56 |
| *Ao1-4-3R* | GCGTCAGTTATTCTCCATCATT |  |
| *Ao1-4-4F* | AATGATGGAGAATAACTGACGC | 56 |
| *Ao1-4-4R* | GTACATCAAGAGAAGCAGTCGA |  |
| *Ao1-4-5F* | GCTAATGATTTGAGATGCAGGA | 56 |
| *Ao1-4-5R* | CAGCCAAAGTGAAGATCAGCAA |  |
| *Ao1-6-1F* | ATGGGACAACCGAACTCATA | 56 |
| *Ao1-6-1R* | TCCCTACGGCTTCTCGACCA |  |
| *Ao1-6-2F* | GTCAGCTCCACTCTTACTTTCA | 55 |
| *Ao1-6-2R* | TGCCCATGCTTTCTTCTATTTG |  |
| *Ao1-6-3F* | ACCTTACCTAGAGTGCTTGTAT | 56 |
| *Ao1-6-3R* | AAACATAAATAGCCCGCATATC |  |
| *Ao2-2-1F* | CGGCTATCACTCTCCTCAGTTCTC | 58 |
| *Ao2-2-1R* | TGCCCCTATATCTTCATGGTCGTG |  |
| *Ao2-2-2F* | CAGCATGGAATGTATGGATGTGAA | 58 |
| *Ao2-2-2R* | CGTTCCTTCTTTAATGGTAGTAGC |  |
| *Ao3-4-1F* | TCTGCGGGAGTTGGTCAATT | 56 |
| *Ao3-4-1R* | CCCACCTCGGATCTCTGCAT |  |
| *Ao3-4-2F* | ATGCAGAGATCCGAGGTGGG | 56 |
| *Ao3-4-2R* | GCTTCTGTTGGCTTCGTTGT |  |
| *Ao3-8-1F* | CCTTCTCCTTTGAACTCTTGCC | 57 |
| *Ao3-8-1R* | TATACCATGATGCCAAAAACCG |  |
| *Ao3-8-3F* | GTGTGGTTCTGGTATACTCTTA | 57 |
| *Ao3-8-3R* | TACTGGAAGACAAGATCTCGAA |  |
| *Ao3-8-4F* | AGATCTTGTCTTCCAGTACCTA | 56 |
| *Ao3-8-4R* | GGTCCAACATAACATATCACGA |  |
| *Ao4-5-1F* | TCTACCCCTAGTCCCAACGTTA | 58 |
| *Ao4-5-1R* | CATGATGACTTCTGACCTGTGC |  |
| *Ao4-5-2F* | TATTCAAGCTCAGGGCACAGGT | 58 |
| *Ao4-5-2R* | TCTTTGAAATGCCGCTGTGTGT |  |
| *Ao4-5-4F* | CCTACTCAAGATCGACTCAACC | 57 |
| *Ao4-5-4R* | GAGAGAAACCCTAACCCTAGCG |  |
| *Ao5-1-1F* | GCCAACATCAGCAGCTAGTACTCCG | 59 |
| *Ao5-1-1R* | TGATGATGGTTCTGGTGTACGCAAC |  |
| *Ao5-1-2F* | GTTGCGTACACCAGAACCATCATCA | 59 |
| *Ao5-1-2R* | ACATTACCACCTTCACAAAATCCCA |  |
| *Ao6-3-1F* | GATCCAACTTCCACAATCTCAC | 57 |
| *Ao6-3-1R* | AGTCAAACCACATGATAGCTCT |  |
| *Ao6-3-2F* | AGAGCTATCATGTGGTTTGACT | 57 |
| *Ao6-3-2R* | TCTGGATTTACCGACATTAGCA |  |
| *Ao7-7-1F* | TCACAGAACTCAGAGGACGATGGAT | 59 |
| *Ao7-7-1R* | TCGCAATGCTCGTCTGGCCTAGAAA |  |
| *Ao7-7-2F* | TCTATATCGTGTTTCTAGGCCAGAC | 58 |
| *Ao7-7-2R* | GATTTACTTCTGATCTGGCTGCTTC |  |
| *Ao8-8-1F* | TGGTGGAGGATAGTAGTGATGTTGG | 59 |
| *Ao8-8-1R* | CAGTGATGATTGGTCTTTCTGCTAC |  |
| *Ao8-8-2F* | ATCATTGTCTCTTCCTTCATCCTCC | 59 |
| *Ao8-8-2R* | AGCATAGATTTGTTCCATCAGAAGC |  |
| *Ao9-3-1-1F* | TTGTCACAGCAGCCAAGTCG | 56 |
| *Ao9-3-1-1R* | GTCTGTCTATTTGGGTGGGT |  |
| *Ao9-3-1-2F* | ACCCACCCAAATAGACAGAC | 57 |
| *Ao9-3-1-2R* | CCAGTACCAACAGTATCGAC |  |
| *Ao9-3-2F* | GTCGATACTGTTGGTACTGGATTGC | 59 |
| *Ao9-3-2R* | TCGCTTCATTGTTATTGGCCTCCTC |  |
| *Ao10-7-1F:* | AGCAGATAAGTTGGTGGGTC | 57 |
| *Ao10-7-1R* | TGAACAAGCATTCCAGAGCA |  |
| *Ao10-7-2F* | TCATGGTTGCACAGACTCAC | 57 |
| *Ao10-7-2R* | GTTAAAGATGCAAGCCCCAG |  |
| *Ao10-7-3F* | GCTTGCATCTTTAACCGTTT | 57 |
| *Ao10-7-3R* | GCAGGCTCCATTCAAGTATC |  |
| *MSY1-F* | TTATCTCTACTTTTGCTCGCTG | 57 |
| *MSY1-R* | AATTCGTCCCTCAAGTGG |  |
| *MSY2-F* | GTCCCGCAAAGCACTTAAAT | 56 |
| *MSY2-R* | TTTCTATGGACGGAAGGGAG |  |
| *MSY3-F* | GTCGCTGTCGCCCTCGTA | 56 |
| *MSY3-R* | AATTCACTGCCAGCTGCTTG |  |
| *MSY4-F* | CTCCTCTACCCAAACAGATCACT | 56 |
| *MSY4-R* | GCTTCGTTTCATGCACCTACAGC |  |
| *MSY5-F* | AAAGGTATAATGTTAAAGTATA | 56 |
| *MSY5-R* | CAGGTTATGGTTAGGACA |  |
| *MSY6-F* | GAGAGGAGATAATCATACACAT | 57 |
| *MSY6-R* | GTGCAACTATATAGGAAAGAAA |  |
| *MSY7-F* | AGAATTAAAGCTACCGGCAT | 56 |
| *MSY7-R* | AGCCCTTGTCAGTTAAAAGA |  |
| *MSY8-F* | AGCGTCTATGATCAAATGCC | 56 |
| *MSY8-R* | CGATTCGCCATTTCTAGGTG |  |
| *MSY9-F* | TCAAGCTCAGGTCAGACAAC | 57 |
| *MSY9-R* | AATGGAATCTGGGAGCTATG |  |
| *MSY10-F* | ATGAATGCTTTGGATCTGGA | 57 |
| *MSY10-R* | CACAACAACACGAGAACCAT |  |
| *MSY11-F* | TTGTAGAATGTCCATCTTGC | 56 |
| *MSY11-R* | CAAAAACTGAATGCGAATCC |  |
| *MSY12-F* | AATAACGAGAGGCACCAAGA | 56 |
| *MSY12-R* | GTGTGCTTCCTTCTCTTCAT |  |
| *MSY13-F* | TTATCCTTCTCTTGGGCAAA | 56 |
| *MSY13-R* | GTTGTTTATCAGCTGCCACC |  |
