# Supplementary material for: Development and applications of a collection of single copy gene-based cytogenetic DNA markers in garden asparagus
Source: Front Plant Sci. 2022 Sep 29;13:1010664. doi: 10.3389/fpls.2022.1010664 (PMC9559582; doi:10.3389/fpls.2022.1010664)
Supplement: Supplementary file 3 [file Table_2.docx]

Table S2. Detailed information of the 71 single-copy genes free of repeats of garden asparagus.

| **Gene ID** | **Gene name** | **Length (bp)** | **Chromosome** | | **Origination** | | **Termination** | |
| --- | --- | --- | --- | --- | --- | --- | --- | --- |
| LOC109846976 | *Ao1-1* | 7684 | 5 | | 5567108 | | 5574791 | |
| LOC109847450 | *Ao1-2* | 5848 | 5 | | 6509204 | | 6503357 | |
| LOC109831664 | *Ao1-3* | 4245 | 5 | | 20892156 | | 20887912 | |
| LOC109825211 | *Ao1-4* | 5772 | 5 | 64286784 | | 64281013 | |  |
| LOC109828887 | *Ao1-5* | 4003 | 5 | 107394229 | | 107390227 | |  |
| LOC109828924 | *Ao1-6* | 4378 | 5 | 109045790 | | 109041413 | |  |
| LOC109829931 | *Ao2-1* | 4574 | 8 | 3202119 | | 3197546 | |  |
| LOC109831691 | *Ao2-2* | 8645 | 8 | 77443879 | | 77452523 | |  |
| LOC109832556 | *Ao3-1* | 4091 | 6 | 702913 | | 698823 | |  |
| LOC109832609 | *Ao3-2* | 9307 | 6 | 1863440 | | 1872746 | |  |
| LOC109832735 | *Ao3-3* | 6168 | 6 | 4997840 | | 5004007 | |  |
| LOC109833085_1 | *Ao3-5* | 4218 | 6 | 13624609 | | 13628826 | |  |
| LOC109834925 | *Ao3-6* | 6884 | 6 | 33637131 | | 33630248 | |  |
| LOC109833863 | *Ao3-7* | 4379 | 6 | 38841857 | | 38846235 | |  |
| LOC109833867 | *Ao3-8* | 5207 | 6 | 38951082 | | 38956288 | |  |
| LOC109835079 | *Ao3-9* | 4897 | 6 | 49423026 | | 49418130 | |  |
| LOC109834220 | *Ao3-10* | 4041 | 6 | 60920710 | | 60924750 | |  |
| LOC109836306 | *Ao4-1* | 5654 | 3 | 1232049 | | 1237702 | |  |
| LOC109836426 | *Ao4-2* | 4847 | 3 | 4898666 | | 4903512 | |  |
| LOC109838351 | *Ao4-3* | 7413 | 3 | 22944311 | | 22936899 | |  |
| LOC109837124 | *Ao4-4* | 6400 | 3 | 32289338 | | 32282939 | |  |
| LOC109837191 | *Ao4-5* | 4683 | 3 | 36621730 | | 36626412 | |  |
| LOC109837363 | *Ao4-6* | 4857 | 3 | 45757654 | | 45752798 | |  |
| LOC109837411 | *Ao4-7* | 4701 | 3 | 49224054 | | 49228754 | |  |
| LOC109837743 | *Ao4-8* | 5002 | 3 | 74402446 | | 74397445 | |  |
| LOC109839310 | *Ao4-9* | 4376 | 3 | 131457997 | | 131453622 | |  |
| LOC109841951 | *Ao5-1* | 8021 | 4 | 27970139 | | 27978159 | |  |
| LOC109843528 | *Ao5-2* | 8861 | 4 | 60651556 | | 60642696 | |  |
| LOC109844065 | *Ao5-3* | 5307 | 4 | 79918800 | | 79924106 | |  |
| LOC109840148 | *Ao5-4* | 4142 | 4 | 91007497 | | 91011638 | |  |
| LOC109840197 | *Ao5-5* | 4299 | 4 | 93069235 | | 93064937 | |  |
| LOC109841106 | *Ao5-6* | 5034 | 4 | 124074390 | | 124069357 | |  |
| LOC109844330 | *Ao6-1* | 4115 | 7 | 3745015 | | 3749129 | |  |
| LOC109844296 | *Ao6-2* | 5609 | 7 | 15676547 | | 15682155 | |  |
| LOC109845972 | *Ao6-3* | 6386 | 7 | 28335011 | | 28341396 | |  |
| LOC109845544 | *Ao6-4* | 4542 | 7 | 30435452 | | 30439993 | |  |
| LOC109848323 | *Ao7-1* | 6164 | 1 | 1757511 | | 1751348 | |  |
| LOC109849872 | *Ao7-2* | 6210 | 1 | 5498933 | | 5505142 | |  |
| LOC109849795 | *Ao7-3* | 6692 | 1 | 8302654 | | 8295963 | |  |
| LOC109849025 | *Ao7-4* | 4325 | 1 | 14967124 | | 14962800 | |  |
| LOC109848491 | *Ao7-5* | 4258 | 1 | 40471121 | | 40466864 | |  |
| LOC109850386 | *Ao7-6* | 4407 | 1 | 123958327 | | 123962733 | |  |
| LOC109848092 | *Ao7-7* | 7316 | 1 | 139581277 | | 139573962 | |  |
| LOC109850652 | *Ao7-8* | 4194 | 1 | 147579923 | | 147575730 | |  |
| LOC109849197 | *Ao7-9* | 5037 | 1 | 151008058 | | 151013094 | |  |
| LOC109819993 | *Ao8-1* | 4806 | 2 | 322343 | | 317538 | |  |
| LOC109851228 | *Ao8-2* | 4514 | 2 | 546454 | | 541941 | |  |
| LOC109820025 | *Ao8-3* | 5900 | 2 | 1246147 | | 1252046 | |  |
| LOC109822173 | *Ao8-4* | 5963 | 2 | 6137130 | | 6131168 | |  |
| LOC109820275 | *Ao8-5* | 5790 | 2 | 10165275 | | 10159486 | |  |
| LOC109819683_1 | *Ao8-6* | 4363 | 2 | 10545067 | | 10540705 | |  |
| LOC109851214 | *Ao8-7* | 4067 | 2 | 53866987 | | 53862921 | |  |
| LOC109851190 | *Ao8-8* | 7168 | 2 | 128118248 | | 128111081 | |  |
| LOC109851518 | *Ao8-9* | 6156 | 2 | 128180929 | | 128187084 | |  |
| LOC109819977 | *Ao8-10* | 4846 | 2 | 128448865 | | 128444020 | |  |
| LOC109822915 | *Ao8-11* | 4548 | 2 | 130741003 | | 130736456 | |  |
| LOC109820387 | *Ao8-12* | 4120 | 2 | 131225436 | | 131229555 | |  |
| LOC109824319 | *Ao9-1* | 4361 | 10 | 2587784 | | 2583424 | |  |
| LOC109823744 | *Ao9-2* | 6170 | 10 | 4640866 | | 4634697 | |  |
| LOC109824012 | *Ao9-3* | 8109 | 10 | 10773569 | | 10781677 | |  |
| LOC109824276 | *Ao9-4* | 5615 | 10 | 21956433 | | 21962047 | |  |
| LOC109823978 | *Ao9-5* | 5037 | 10 | 63909878 | | 63914914 | |  |
| LOC109824412 | *Ao9-6* | 5498 | 10 | 65058904 | | 65053407 | |  |
| LOC109824472 | *Ao9-7* | 4609 | 10 | 68057476 | | 68052868 | |  |
| LOC109825379 | *Ao10-1* | 4259 | 9 | 601189 | | 596931 | |  |
| LOC109824912_1 | *Ao10-2* | 4436 | 9 | 2320924 | | 2325359 | |  |
| LOC109825562 | *Ao10-3* | 4076 | 9 | 9854300 | | 9850225 | |  |
| LOC109825344 | *Ao10-4* | 4165 | 9 | 60014871 | | 60019035 | |  |
| LOC109825574 | *Ao10-5* | 4074 | 9 | 62172505 | | 62168432 | |  |
| LOC109825143 | *Ao10-7* | 8226 | 9 | 72435441 | | 72443666 | |  |
| LOC109825232 | *Ao10-8* | 4697 | 9 | 73167419 | | 73172115 | |  |

Note: the gene name *Ao1-1* represents this gene located on the chromosome 1 of the genome assembly. The ‘chromosome’ denotes the probes are localized on the specific chromosome of karyotypic analysis.
