# Supplementary material for: Development and applications of a collection of single copy gene-based cytogenetic DNA markers in garden asparagus
Source: Front Plant Sci. 2022 Sep 29;13:1010664. doi: 10.3389/fpls.2022.1010664 (PMC9559582; doi:10.3389/fpls.2022.1010664)
Supplement: Supplementary file 4 [file Table_3.docx]

Table S3. The blocking DNA gradient exploration system.

| Experimental system | 1 | 2 | 3 | 4 |
| --- | --- | --- | --- | --- |
| Blocking genome DNA/Probe | (0) | (3:1) | (6:1) | (9:1) |
| Blocking genome DNA (amount/volume) | 0 | 3000 ng/2 µL | 6000 ng/4 µL | 9000 ng/6 µL |
| Probe (amount/volume) | 1000 ng/3.5 µL | 1000 ng/3.5 µL | 1000 ng/3.5 µL | 1000 ng/3.5 µL |
| 45S rDNA (volume) | 0.5 µL | 0.5 µL | 0.5 µL | 0.5 µL |
| 2×SSC + 1×TE buffer (volume) | 6 µL | 4 µL | 2 µL | 0 µL |
